# The anti-inflammatory and anti-glycative effects of rosmarinic acid in the livers of type 1 diabetic mice

**DOI:** 10.1051/bmdcn/2017070319

**Published:** 2017-08-25

**Authors:** Yu-Ju Wen, Mei-Chin Yin

**Affiliations:** 1 Department of Nutrition, China Medical University Taichung 404 Taiwan

**Keywords:** Rosmarinic acid, Diabetes, RAGE, PGE_2_

## Abstract

Background: Rosmarinic acid (RA) is a polyphenol present in members of the *Lamiaceae* family. In this study, yhe anti-inflammatory and anti-glycative effects of RA in the livers of type 1 diabetic mice were examined.

Methods: The diabetic mice were divided into three groups: diabetic mice with 0, low dose RA (25 mg/*ml*), and high dose RA (50 mg/*ml*). One group of non-diabetic mice was used as a control for comparison. RA was supplied *via* daily 200 μL oral injections for 9 weeks. The level of interleukin (IL)-6, the tumor necrosis factor (TNF)-alpha, the prostaglandin E_2_ (PGE_2_), and the activity of cyclooxygenase (COX)-2 in the livers were measured. The hepatic receptor of advanced glycative endproduct (RAGE), the sorbitol levels, and the glyoxalase 1 (GLO-1) activity were also determined.

Results: Compared with diabetic group that received no RA, the groups with RA supplements at both levels of dosages had increased body weight and had both decreased water intake and feed intake (*p* < 0.05). RA intake was found to reduce plasma glucose level and elevate plasma insulin level when compared with the diabetic group that received no RA (*p* < 0.05). RA treatments lowered the hepatic level of IL-6, TNF-alpha, and PGE_2_, as well as the activity of COX-2 (*p* < 0.05). RA administration also decreased hepatic RAGE and sorbitol levels, and GLO-1 activity when compared with the diabetic group that received no RA (*P* < 0.05).

Conclusion: These findings support the conclusion that rosmarinic acid (RA) could be a potent protective agent for the liver against diabetic injury.

## 1. Introduction

Rosmarinic acid (RA) is a main polyphenol present in *Rosmarinus Officinalis* L., Coleus aromaticus, and members of the Lamiaceae family. It has been documented that RA has many bio-activities including anti-oxidative, anti-microbial, anti-inflammatory, anti-metastatic, neuroprotective, and immunomodulatory effects [1–3]. Furthermore, RA has been considered to be a potent agent for chronic disease prevention and/or alleviation [3–5]. The anti-diabetic effects of RA in rodents have been examined, and the authors of those studies have indicated that RA could improve glycemic control, oxidative stress and vascular dysfunction, which in turn can attenuate the progression of diabetes, as well as delay the occurrence of diabetic complications [6–8].

Inflammation and glycation are two major pathological characteristics of diabetes types 1 and 2. Although the antiinflammatory effect of RA has been reported [9], it is still unclear whether RA intake could alleviate hepatic inflammation under diabetic conditions. The liver preserves many nutrients and exerts many crucial physical functions. If RA does protect the liver against diabetes-related inflammation, it may be a beneficial nutritional support for diabetic subjects. Thus, in our present study we examined the impact of RA upon a variety of inflammatory factors including interleukin (IL)-6, prostaglandin E_2_ (PGE_2_), and cyclooxygenase (COX)-2 in the livers of type 1 diabetic mice to evaluate the anti-inflammatory effects of RA. Thus far, less attention has been paid to the anti-glycative activities of RA, especially in the liver. The increase in the receptors of advanced glycative endproducts (RAGE) plays an important role in diabetic progression because RAGE reacts with AGEs and other ligands such as beta-amyloid, and the interactions of RAGE and its ligands further activates other signal pathways responsible for the production of oxidative, inflammatory, or angiogenic factors [10–12]. Thus, the decline of RAGE formation due to RA definitely contributes to diminishing glycative injury and other diabetic pathological stress. In addition, sorbitol level and glyoxalase 1 (GLO-1) activity are two biomarkers used for evaluating glycative stress. If RA decreases sorbitol generation and/or increases GLO-1 activity, it subsequently may alleviate glycative injury. In our present study, type 1 diabetes was induced in mice, and followed with RA treatment. The anti-inflammatory and anti-glycative effects of RA in the livers of these mice was examined.

## 2. Materials and Methods

### 2.1. Materials

RA (97%), propylene glycol (PG, 98%), and streptozotocin (STZ, 99.5%) were purchased from Sigma Chemical Co. (St. Louis, MO, USA). RA was dissolved in PG at 50 mg*/ml.*

### 2.2. Animals and diet

Male Balb/c mice at 5-wk old were obtained from the National Laboratory Animal Center (Taipei City, Taiwan). The mice were housed in a 12-h light and 12-h dark cycle. A standard diet and water were supplied ad libitum. Mice with a body weight of 24.2 ± 1.6 g were selected to be induced with diabetes by a single IV injection of STZ at 50 mg/kg into the tail vein after mice had been fasting for 12 h. At day 10, the blood glucose level of 12-h fasted mice was measured *via* a one-touch blood glucose meter (Lifescan Inc., Milpitas, CA, USA). Mice with a blood glucose level ≥ 200 *mg/dl* were used. Use of those mice was approved by the China Medical University Animal Care and Use Committee, and the permission number was 104-305.

### 2.3. Animal Experiment

The diabetic mice were divided into three groups: diabetic mice with 0, low dose RA (25 mg/*ml*), and high dose RA (50 mg/ *ml*). One group of non-diabetic mice was used as a control for comparison. RA at 200 μL was supplied every day *via* oral injection. Body weight and glucose level were recorded every week. After a 9-week supplementation, mice were fasted for 12 h and then killed with carbon dioxide. Both blood and liver from each mouse were collected. The protein concentration of liver homogenate was measured by a commercial kit (Pierce Biotechnology Inc., Rockford, IL, USA).

### 2.4. Insulin analysis

Plasma insulin level (μg/L) was assayed by an insulin radioimmunoassay kit purchased from Linco Research Inc. (St. Charles, MO, USA).

### 2.5. Measurement of inflammatory factors

The hepatic levels of IL-6 and tumor necrosis factor (TNF)-alpha were measured by using cytoscreen immunoassay kits (BioSource International, Camarillo, CA, USA). The PGE_2_ level and COX-2 activity were determined by kits purchased from Cayman Chemical Co. (Ann Arbor, MI, USA). The COX-2 activity was assayed by monitoring the variation of absorbance at 590 nm, which indicated the generation of oxidized N, N, N’, N’-tetramethyl-p- phenylenediamine.

### 2.5. Determination of glycative factors

The hepatic RAGE and sorbitol levels were determined by a colorimetric assay kit (BioVision, Mountain View, CA, USA) and a SEA645Mu ELISA kit (USCNK Life Science Inc., Hubei, China), respectively. The GLO-1 activity was quantified by a commercial kit obtained from Bioassay Co. (Hayward, CA, USA).

### 2.6. Statistical analyses

Data were expressed as mean ± standard deviation (SD). Each group had eight mice (n = 8). Statistical analysis was processed by using a one-way analysis of variance, and Dunnett’s t-test was used for *Post-hoc* comparisons. A *P* value < 0.05 was defined as significant.

## 3. Results

**Fig. 1:**
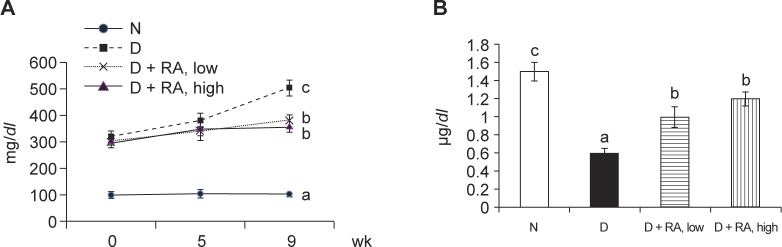
Plasma glucose level (mg/*dl*, a) and plasma insulin level (μg/*l*, b) in normal (N) or diabetic mice (D) treated with RA at 0, low, or high dose for 9 weeks. Values are mean ± sd, n = 8. ^a-c^means among bars without a common letter differ, *p* < 0.05.

**Fig. 2:**
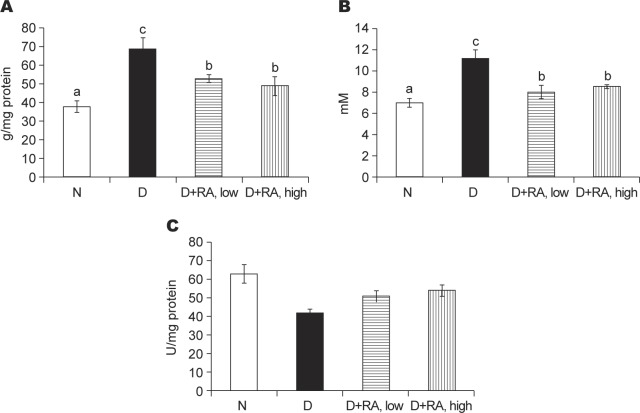
The hepatic level of RAGE (μg/mg protein, a) and sorbitol (mM, b), and GLO-1 activity (U/mg protein, c) in normal (N) or diabetic mice (D) treated with RA at 0, low, or high dose for 9 weeks. Values are mean ± SD, n = 8. ^a-e^means among bars without a common letter differ, *p* < 0.05.

Compared with the diabetic group of mice that received no RA, RA supplements at both low and high doses increased body weight, and decreased water intake and feed intake (Table 1, *p* < 0.05). As shown in Fig. 1, RA intake reduced the plasma glucose level (a), and elevated the plasma insulin level (b) when compared with the diabetic group of mice that received no RA (*p* < 0.05). RA supplements reduced the hepatic level of IL-6, TNF-alpha and PGE_2_, and the activity of COX-2 (Table 2, *p* < 0.05). As shown in Fig. 2, RA intake lowered the hepatic level of RAGE (a) and sorbitol (b), and raised the GLO-1 activity (c) when compared with the diabetic group of mice that received no RA (*p* < 0.05).

**Table 1:** body weight (BW), water intake (WI, *ml/*mouse/day), feed intake (FI, g/mouse/day) in normal (N) or diabetic mice (D) treated with RA at 0, low, or high dose for 9 weeks. Values are mean ± SD, n = 8. ^a-c^means in a row without a common letter differ statistically *p <* 0.05.

	N	D	D + RA, low	D + RA, high
BW	32.4 ± 0.9^c^	20.1 ± 1.1^a^	23.5 ± 0.6^b^	22.7 ± 0.8^b^
WI	2.3 ± 0.3^a^	6.5 ± 1.0^c^	5.1 ± 0.5^b^	4.5 ± 0.7^b^
FI	2.1 ± 0.5^a^	6.8 ± 0.8^c^	5.3 ± 0.6^b^	4.7 ± 0.4^b^

**Table 2:** The hepatic level of IL-6 (pg/mg protein), TNF-alpha (pg/mg protein), PGE_2_ (μg/mg protein), and COX-2 activity (nmol/min/mg protein) in normal (N) or diabetic mice (D) treated with RA at 0, low, or high dose for 9 weeks. Values are mean ± SD, n = 8. ^a-d^means in a row without a common letter differ statistically, *p* < 0.05.

	N	D	D + RA, low	D + RA, high
IL-6	40 ± 5^a^	103 ± 9^b^	54 ± 8^a^	46 ± 4^a^
TNF-alpha	4.8 ± 0.6^a^	8.3 ± 1.0^b^	5.2 ± 0.3^a^	5.1 ± 0.5^a^
PGE_2_	6.4 ± 0.4^a^	19.5 ± 1.1^c^	14.4 ± 0.7^b^	12.9 ± 0.9^b^
COX-2	1.8 ± 0.2^a^	16.8 ± 0.8^d^	11.1 ± 1.0^c^	5.7 ± 0.6^b^

## 4. Discussion

As reported by others [6–8] and observed by our present study, RA supplement attenuates the pathological progression of diabetes, which is evidenced by decreased water and feed intake, lowered blood glucose level, and increased insulin level. Furthermore, we found that RA intake effectively alleviated hepatic inflammatory and glycative stress *via* decreasing the production or activity of inflammatory and glycative factors such as IL-6, TNF-alpha, PGE_2_, COX-2, RAGE and sorbitol, and increasing the GLO-1 activity. These novel findings support the notion that RA protects the liver against inflammatory and glycative injury from diabetes, which in turn benefits the hepatic functions.

Both IL-6 and TNF-alpha are inflammatory cytokines and are commonly used as biomarkers for evaluating inflammatory status, especially under a diabetic condition [13]. The results of our present study indicate that RA treatments effectively decrease the hepatic release of IL-6 and TNF-alpha. These data support the anti-inflammatory activity of RA and show that the anti-inflammatory protection of RA extends to the liver under a diabetic condition. In addition, COX-2 and PGE_2_, which is a metabolite of COX-2, are important pathological mediators that contribute to the progression of inflammatory disorders including diabetes [14, 15]. Thus, the decline in COX-2 activity and PGE_2_ formation due to RA treatments observed in our present study suggest the attenuation in hepatic inflammatory stress. These results also explain the anti-inflammatory actions of RA that were observed.

The pathological importance of RAGE has attracted more attention because RAGE can bind many ligands including AGEs, high-mobility group box protein 1, β-amyloid and even lipopolysaccharide [16, 17]. Our present study found that RAGE production in the liver becomes elevated due to diabetes. It is possible that the increased RAGE in the liver not only favors glycative reactions by reacting with AGEs, but also promots inflammatory and/or oxidative response by reacting with other ligands. Our data revealed that RA treatments at both low and high doses lowered hepatic RAGE formation, which subsequently decreased the interaction between RAGE and its ligands, and finally they mitigated RAGE-associated diabetic pathological development. The polyol pathway is responsible for the conversion of glucose to sorbitol through the action of aldose reductase [18]. The activation of this pathway facilitates the pathogenesis of diabetic microvascular complications because the excessive sorbitol and its downstream products promote AGEs formation and enhance glycative stress [19, 20]. In our present study, an increased hepatic sorbitol level was found, which implies that the polyol pathway was activated in the livers of the diabetic mice. However, RA treatments at both low and high doses markedly reduced hepatic sorbitol generation. It is thus possible that RA limited aldose reductase activity in the livers of diabetic mice, which consequently suppressed sorbitol production and diminished hepatic glycative stress. On the other hand, it is known that glycative products and their precursors that are formed due to diabetic stress can be metabolized by the glyoxalase system to less toxic compounds, in which the glycative injury or stress can be attenuated [21, 22]. GLO-1 is the rate-limiting enzyme of the glyoxalase system, and it is considered to be an important anti-glycation enzyme because the enhancement of this enzyme enhances the host’s defensive capability against glycation [23]. Thus, the raised hepatic GLO-1 activity from RA treatments observed in our present study might be responsible for contributing to a decrease in the production of hepatic glycative products and their precursors. These results suggest that RA may ameliorate glycative injuries in the liver under a diabetic condition through regulating polyol and glyoxalase pathways.

In summary, a supplementation of rosmarinic acid (RA) decreased the inflammatory and glycative stress in the livers of diabetic mice *via* decreasing the generation of IL-6, TNF-alpha, PGE_2_, RAGE, and sorbitol, as well as reducing COX-2 activity and raising GLO-1 activity. Such findings support that this polyphenol could be considered as a potent protective agent for the liver against diabetic injury.
